# Ageism among postgraduate medical trainees: A mixed-method study from Karachi, Pakistan

**DOI:** 10.12669/pjms.42.6.15807

**Published:** 2026-06

**Authors:** Aayesha Butt, Saniya Sabzwari, Munazza Asad, Anny Dhanwani

**Affiliations:** 1 Aayesha Butt, *FCPS (Family Medicine)*, Department of Family Medicine, Dow University of Health Sciences, Karachi, Pakistan; 2 Saniya Sabzwari, *MD, MHPE, Fellow AAFP*, Aga Khan University Hospital, Karachi, Pakistan; 3 Munazza Asad, *FCPS (Family Medicine)*, Aga Khan University Hospital, Karachi, Pakistan; 4 Anny Dhanwani, *FCPS (Community Medicine)*, Aga Khan University Hospital, Karachi, Pakistan

**Keywords:** Ageism, Attitudes, Postgraduate Trainees, Healthcare Professionals, Older Adults, Geriatric Care, Mixed-Methods

## Abstract

**Background & Objectives:**

Ageism - stereotyping, prejudice, and discrimination against older adults – can negatively impact health care delivery. In Pakistan, the aging population is growing yet little is known about the attitudes of postgraduate medical trainees towards older patients. The objective was to assess the prevalence of ageist attitudes among postgraduate trainees in Karachi and explore underlying perceptions influencing these views.

**Methodology:**

A convergent mixed-method cross-sectional study was conducted from September 2022 till September 2023 in Karachi, Pakistan, among trainees enrolled in CPSP programs. Quantitative data were collected using modified University of California, Los Angeles (UCLA) Geriatric Attitude Scale, and qualitative insights were obtained through two focus group discussions (FGDs). Descriptive statistics and logistic regression analyzed survey data, while thematic analysis explored FGD transcripts.

**Results:**

Of 218 respondents (mean age 29 years, 74% women), 88% frequently encountered older patients, yet 55% found their care challenging. The median attitude score was 45/65, with 46% scoring above the median. Trainees who opposed geriatric education or government-run geriatric centers were significantly three times more likely to exhibit ageist attitudes (p<0.05). FGDs revealed limited awareness of ageism, perceived complexity of geriatric care due to multimorbidity and communication barriers, and strong support for capacity building through structured education and infrastructure.

**Conclusion:**

Despite frequent geriatric encounters, ageist attitudes persist among trainees, driven by inadequate training and systemic constraints. Integrating standardized geriatric education and establishing dedicated care facilities are essential to improve attitudes and enhance care for older adults.

## INTRODUCTION

Ageism, defined as stereotyping, prejudice, and discrimination towards older adults, is comparable to sexism creates barriers to both the quantity and quality of care.^1^ The World Health Organization identifies ageism as a barrier to healthy aging, affecting individuals, systems and society, including patient self-perception.^2^

Older adults account for approximately a quarter of the global disease burden and frequently access healthcare services; however, millions face neglect, underdiagnosis and abuse. Ageism contributes significantly to poor outcomes, including an estimated 6.3 million cases of depression worldwide.^3^

Healthcare professionals often view older adults through a lens of frailty and decline, reinforcing negative stereotypes that affect care quality.^4^ In Pakistan, where approximately 12.5 million people are aged 60 or above, most face chronic illnesses and limited geriatric services.^5^ While capacity-building initiatives to enhance knowledge and skills have begun, postgraduate trainees’ attitude towards older adults remain largely unexplored.^6^

This study aimed to address this critical gap by assessing the prevalence of ageist attitude among postgraduate medical trainees and exploring the perception underlying these attitudes. Understanding this attitude is essential for informing educational reforms and policy interventions that promote age-inclusive, compassionate, and competent care for the country’s growing elderly population.

### Objectives:

1. To explore the presence of ageist attitudes among postgraduate medical trainees in Karachi, Pakistan.
2. To identify and analyze their underlying perceptions and beliefs towards caring for older adults.

## METHODOLOGY

The study employed a convergent mixed-method design, in which quantitative survey data and qualitative findings from focus group discussions were collected during the same phase and analyzed separately. The findings from both components were then integrated during interpretation to provide a comprehensive understanding of trainees’ attitudes toward older adults. Ageist attitudes among postgraduate medical trainees in Karachi registered under CPSP were assessed. Medical officers, students of diploma or master’s programs and trainees who had graduated more than 10 years ago were excluded. The study was conducted from September 2022 till September 2023 in Karachi, Pakistan.

### Ethical approval:

It was obtained from the Institutional Review Board of Aga Khan University Hospital, Pakistan (Study ID: 2022-7545-23260; Dated: November 2, 2022).

The questionnaire was distributed as a Google Form via email and WhatsApp, with an electronic informed consent form attached. A convenience sampling strategy with required sample size was calculated using Open-Epi for a population of 5,000, an expected frequency of 44%, 7 a 95% confidence level, and a margin of error of 6.5%, resulting in an estimated sample size of 215 participants.

The questionnaire captured basic demographic information, interaction, and training related to geriatrics. The validated University of California, Los Angeles (UCLA) Geriatrics Attitudes Scale was used with permission.^8^ It is a 14 items Likert scale developed to assess health care providers’ attitudes toward older persons. One question “*federal government should reallocate money from Medicare to research on AIDS or pediatric diseases*”—was removed because it refers to the Medicare system, which is not applicable to the Pakistani healthcare context. After removal of this item, the modified instrument consisted of 13 items, each scored on a five-point Likert scale (1 = strongly disagree to 5 = strongly agree). Negatively worded items were reverse scored according to the original scoring instructions so that higher total scores reflected more positive attitudes toward older adults.

The total possible score for the modified scale (calculated by summing the scores of all items) ranged from 13 to 65. Because no validated cut-off exists to define ageist attitudes using this scale, the sample median score was used to categorize participants into relatively less positive (<45) and more positive (≥45) attitude groups. The modified scale was piloted on Family Medicine faculty and medical officers to ensure clarity and contextual relevance, Cronbach’s alpha, which demonstrated acceptable reliability (α = 0.70).

Data was collected through two online focus group discussions (FGDs) conducted via Zoom, each involving ten participants from either private or public sector practice (total n=20). Sessions were recorded for transcription and analysis. A semi-structured interview guide was developed to explore participants’ experiences and perspectives related to ageism in clinical practice. Open-ended questions probed their understanding of ageism, encounters with geriatric patients, emotional responses, perceived barriers, and ideas for improvement. Each session was facilitated by a moderator independent of the research team, who possessed prior experience in qualitative research. A second researcher was present to take notes and manage the recording. All sessions were transcribed verbatim in English, de-identified to ensure confidentiality, and transcripts were made available to participants for member checking. Data was analyzed using thematic analysis.

Two researchers independently reviewed the transcripts and conducted open coding to identify recurring concepts, which were then grouped into preliminary codes. Through iterative discussion, these codes were organized into broader themes and sub-themes. Thematic analysis focused on four key areas: participants’ understanding of ageism, its perceived impact on the care of older adults, how clinical encounters with geriatric patients shape physicians’ attitudes, and potential strategies to address ageism. In cases of disagreement between coders, consensus was reached after repeated review of the transcript

### Data analysis:

Data was analyzed using STATA 16. Descriptive statistics were calculated. Associations between variables were assessed using chi-square, followed by binary logistic regression, with attitude score category (<45 vs ≥45) as the dependent variable and sociodemographic and training characteristics as independent variables. A p-value <0.05 was considered statistically significant. Qualitative data were analyzed using thematic analysis, as described in methodology, and the findings were subsequently compared with the quantitative results during interpretation.

## RESULTS

The mean participant age was 29 years (SD ±2.7), and 74% were women. Most were from private institutes (74%) and medical specialties (57%). Majority 82% reported having older adults at home and 79.7% were aware of common geriatric illnesses (Table-I). UCLA ageism scores ranged from 18 to 63, With higher scores indicating more positive attitude towards older adults (median of 45); 46% scored above the median.

**Table I:** Socio-demographic characteristics of Postgraduate trainees (n=218).

Characteristics	Frequency (n)	Percentage (%)
Age	Mean ±SD (29±2.7)	
***Gender***		
Male	57	26
Female	161	74
***PG year***		
PG-1	49	22
PG-2	37	17
PG-3	34	16
PG-4	47	22
PG-5	42	19
PG-6	9	4
***Specialty***		
Medicine	125	57
Surgery	93	43
***Training institute***		
Public	56	26
private	162	74
***Marital status***		
yes	179	82
no	39	18
***Do you have any individual 60 years of age or older in your home?***		
Yes	179	82
No	39	18
***Are you aware of common health problems in older adults?***		
Yes	212	97
No	6	3

**PG:** postgraduate.

Most participants (88%) reported frequent encounters with older patients. Nearly half (45%) found their care challenging, while 88% supported inclusion of geriatric education in training, and 92% favored establishing government-run geriatric centers. Although 90% acknowledged societal responsibility toward older adults and reported greater sympathy for them, 68% preferred treating younger patients. Additionally, 70% perceived geriatric care as resource-intensive and associated older age with disorganization and confusion (Table-II).

**Table-II:** Geriatrics attitudes of Postgraduate trainees (n=218)

Items	Disagree n (%)	Neutral n (%)	Agree n (%)
Most old people are pleasant to be with.	36 (16%)	72(33%)	110 (50%)
It is society’s responsibility to provide care for its elderly persons.	14 (6%)	24(11%)	180 (82%)
Elderly patients tend to be more appreciative of the medical care I provide than younger patients.	33 (15%)	45(21%)	140 (64%)
I tend to pay more attention and have more sympathy towards my elderly patients than my younger patients	17 (8%)	59(27%)	142 (65%)
It is interesting listening to old people’s accounts of experience	15 (7%)	35(16%)	168 (77%)
If I have the choice, I would rather see younger patients than elderly ones.	69 (32%)	70(32%)	79 (36%)
Medical care for old people uses up too many human and material resources.	67 (30%)	56(26%)	95 (43%)
As people grow older, they become less organized and more confused.	51 (23%)	53(24%)	114 (52%)
Taking a medical history from elderly patients is frequently an ordeal.	37 (17%)	64(29%)	117 (53%)
Old people, in general, do not contribute much to society	141 (65%)	36(17%)	41 (18%)
Treatment of chronically ill old patients is hopeless.	126 (58%)	54(25%)	38 (17%)
Old people don’t contribute their fair share towards paying for their health care.	124 (57%)	53(24%)	41 (19%)
In general, old people act too slowly for modern society	105 (49%)	57(26%)	56 (26%)

The association between participant characteristics and attitudes scores towards older adults is presented in Table-III. Gender, training institute, older adults at home, years of training and postgraduate specialty were found insignificant as the confidence intervals and p-values were not statistically significant.

**Table III:** Association of socio-demographic characteristics, postgraduate training predictors, and ageism score of health care professionals (n=218).

Variable	Attitude Score <45 n (%)	Attitude Score ≥45 n (%)	Odds Ratio (95% CI)	p-value
***Gender***				
Female	38 (51%)	36 (49%)	1.4 (0.62–3.1)	0.40
Male	16 (62%)	10 (38%)	Ref	
***Training Institute***				
Private	41 (55%)	33 (45%)	1.12 (0.83–1.5)	0.44
Public	13 (50%)	13 (50%)	Ref	
***Postgraduate Training Duration***				
<3 years	31 (56%)	24 (44%)	1.16 (0.71–2.00)	0.45
≥3 years	23 (51%)	22 (49%)	Ref	
***Older adult at home***				
No	11 (61%)	7 (39%)	1.4 (0.7–2.9)	0.30
Yes	43 (52%)	39 (48%)	Ref	
***Specialty***				
Medicine	33 (58%)	24 (42%)	1.2 (0.9–1.6)	0.14
Surgery	21 (49%)	22 (51%)	Ref	
***Frequent encounters with older patients***				
Disagree	7 (56%)	5 (44%)	1.0 (0.47–2.0)	0.80
Agree	47 (54%)	40 (46%)	Ref	
***Care of older patients is easy***				
Disagree	27 (60%)	18 (40%)	1.5 (0.8–2.7)	0.14
Agree	27 (49%)	28 (51%)	Ref	
***Geriatric education should be part of training***				
Disagree	9 (77%)	3 (23%)	3.4 (1.26–9.07)	0.015*
Agree	45 (51%)	43 (49%)	Ref	
***Government geriatric centers should exist***				
Disagree	6 (75%)	2 (25%)	2.9 (1.0–9.4)	0.05*
Agree	48 (52%)	44 (48%)	Ref	

Trainees who disagreed that geriatric education should be included in training was significantly more likely to have lower attitudes score ((OR 3.4; 95% CI 1.26–9.07; p=0.015). Similarly, those who disagreed that government-run geriatric centres should be established, had higher odds of lower attitude scores (OR 2.9; 95% CI 1.0–9.4; p=0.05).

**Fig.1:**
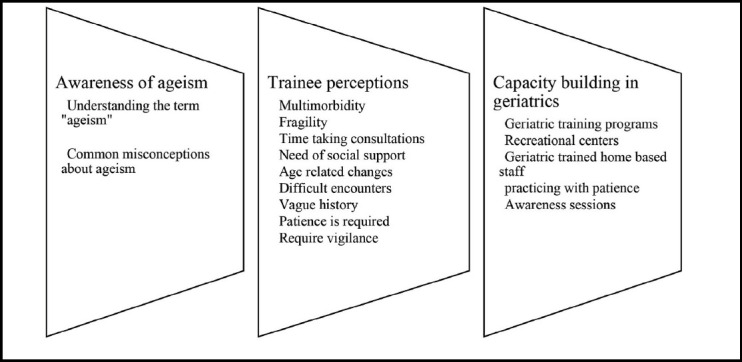
Themes and the subthemes of the FGD.

These qualitative themes provided deeper insight into the quantitative findings by explaining the challenges trainees face when caring for older adults. Three major themes emerged, with responses categorized as shown in Fig.1.

### Awareness of ageism:

Participants from private hospitals were familiar with the term ageism, one stated, “*Discrimination against people based on their age, like treating them differently as compared to others*.” Participants from public hospitals were less familiar, with one noting, “*Ageism is associated with elderly individuals and their ailments*.”

### Perception of geriatric care:

Participants reported multiple challenges in geriatric care, including multi-morbidity and age-related changes such as decreased mobility, hearing, and comprehension. One participant remarked, “*They require lots of patience as they may have a hearing problem, they may use or forget to use hearing aids, they may have difficulty in comprehending instructions … and physicians generally consider these symptoms as age-related*.” Older patients were perceived as fragile, requiring careful attention; “*even a simple cannulation has to be done very carefully*.”

**Supplementary Table:** Characteristics of postgraduate training of participants (n=218)

Characteristics	Disagree	Neutral	Agree
I often encounter older patients in my clinical practice?	27 (12%)	30(14%)	161 (74%)
I find that taking care of these patients is easy	99 (45%)	78(36%)	41 (19%)
I believe geriatric education should be part of my training	26 (12%)	24(11%)	168 (77%)
I believe that there should be some central government centers dedicated to geriatric population.	17 (7.8%)	15(6.9%)	186 (85.3%)

### Capacity building in geriatrics:

Trainees expressed apprehension, with one remarking, “*It provokes a lot of anxiety among physicians, and they can miss gathering some important information*.” A tendency to attribute symptoms to aging was also reported: “*The physicians are also brushing off many symptoms as age-related changes, which can lead to mismanagement*.” Participants emphasized the need for patience and empathy, recommending educational initiatives such as seminars, geriatric fellowships, certificate courses, and diplomas. They also highlighted the importance of recreational centers and staff training for geriatric home visits.

## DISCUSSION

This study highlights a mixed pattern of attitudes towards older adults among postgraduate medical trainees in Karachi. While many trainees expressed positive perception of older adults, some negative attitudes were also observed in both the quantitative and qualitative findings. Older adults face higher health risks and hospital visits, hence important to explore healthcare professionals’ attitudes toward this population. 9 Although many had personal ties to older adults, unfortunately, it did not reduce ageism, possibly due to compartmentalization of experiences.^10^,^11^

Survey responses indicated generally positive attitudes towards older adults, such as recognition of societal responsibility and appreciation for older patients experience. However, many trainees still perceive geriatric care as challenging. The FGD helped explain this discrepancy, highlighting practical barriers such as multimorbidity, communication difficulties, time intensive consultations and limited geriatric training. Thus, the qualitative data suggests that perceived complexity of geriatric care, rather than purely negative attitude towards older adults, may contribute to observed preferences for treating younger patients.

Many trainees felt unprepared to manage complexities such as polypharmacy and cognitive decline. Inadequate training fosters frustration, burnout, and ageist attitudes, with geriatric care often perceived as challenging and less rewarding.^12^ A similar burden of emotional strain among healthcare trainees in Pakistan has been reported, including compassion fatigue, which further impacts attitudes towards patient care.^13^

Participants expressed appreciation for older patients, valuing their life stories, wisdom, and empathy.^14^ Cultural values emphasizing respect for elders further reinforced these positive attitudes, with most respondents believing that society should care for them. In Pakistan, older adults are traditionally esteemed as family leaders whose wisdom guides important decisions.^15^ However, many participants still preferred treating younger patients, highlighting the challenges of geriatric care. This preference may not directly reflect ageism but underscores the importance of capacity building in this field. 16 Notably, over 30% of geriatricians chose the specialty later in their careers.^17^

Gender differences in ageist attitudes were minimal, possibly due to shared training and clinical exposure, though literature suggests women may show more empathy.^18^ Empathy can diminish in professional settings due to standardized education.^19^ Institutional culture also influences attitude: private institutions prioritize efficiency, while public institutions face resource constraints.

In our study, younger professionals showed more positive attitudes. These attitudes declined with experience, likely due to clinical responsibilities and challenges in caring for older patients rather than the patients themselves.^20^ Medicine and allied trainees showed slightly more positive attitudes than surgical trainees, likely due to more frequent interactions with chronic conditions. This is concerning as surgeons’ negative attitudes have been linked to higher surgical risks^21^

The qualitative findings echoed these attitudes. They also revealed limited awareness of ageism, particularly in public-sector institutions. Participants stressed improving infrastructure, training, and resources to address challenges like multimorbidity, cognitive decline, and sensory impairments.^22^ Formal geriatric education was strongly supported, as it reduces ageist attitudes and improves care quality. In Pakistan, where geriatric medicine remains underdeveloped, such initiatives are essential to meet the needs of a rapidly aging population.

### Limitations:

The cross-sectional design of this study limits causal inference and self-reported data may reflect bias. Conducting FGDs via Zoom may have limited participant engagement. Nonetheless, the mixed-method approach strengthens the validity.

## CONCLUSION

Postgraduate trainees held ambivalent attitudes toward older patients, combining sympathy with avoidance, shaped by complex care demands, communication barriers, and insufficient training. These findings highlight persistent ageist tendencies that coexist with trainees’ acknowledged societal responsibility for older adults.

### Recommendations:

Further research is needed to guide training and policy reforms. The use of a median split to categorize attitude scores may result in some loss of information compared with analyzing the scale as a continuous variable; however, this approach allowed clearer interpretation of factors associated with relatively lower versus higher attitude scores in logistic regression analysis.

### Authors Contribution:

**SS and AB:** conceived and designed the study.

**AB and MA:** conducted data collection and qualitative interviews.

**AD:** performed statistical analysis.

**AB, MA, and AD:** drafted the manuscript.

**SS, AB, MA, and AD:** reviewed and edited the manuscript.

**SS:** supervised the study and is responsible for the integrity of the research.
